# Comparison of Some Molecular Markers for Tick Species Identification

**Published:** 2019-06-24

**Authors:** Eman M. Abouelhassan, Hamdy M. El-Gawady, Ahmad Anwar Abdel-Aal, Amal K. El-Gayar, Maria D Esteve-Gassent

**Affiliations:** 1 Department of Veterinary Parasitology, Suez Canal University, Ismailia, Egypt; 2 Department of Veterinary Pathobiology, Texas A and M University, College Station, Texas, United States of America

**Keywords:** Ticks, *Dermacentor*, *Amblyomma*, *Rhipicephalus*, *Ixodes*

## Abstract

**Background:**

Ticks are obligate blood-sucking ectoparasites of vertebrates. Since many tick identification studies are based on the analysis of 16S rDNA, 12S rDNA and ITS-1, 2 rDNA genes, we aimed to compare the performance of these molecular markers of common use for the identification of ticks, under a diagnostic laboratory environment.

**Methods:**

Overall, 192 tick specimens were collected through the state of Texas from January 2014 to August 2015 and the species was determined by both morphology and molecular amplification using the 16S rDNA, 12S rDNA, ITS1 and ITS2.

**Results:**

The species collected were identified by molecular techniques as *Dermacentor albipictus*, *D. variabilis*, *Amblyomma americanum*, *Ixodes scapularis*, *A. cajennense*, *Rhipicephalus sanguineus* and *Carios capensis.* ITS1 and ITS2 were not able to prove consistent amplification and therefore have been considered as potential markers for tick identification.

**Conclusion:**

The use of mitochondrial genes in tick identification showed to provide more consistent results in the diagnostic environment.

## Introduction

Ticks are obligate blood-sucking ectoparasites of vertebrates, causing great economic losses to livestock with its direct and indirect effects on hosts. Bloodsucking by large numbers of ticks causes a reduction in live weight and anemia among domestic animals, while their bites also reduce the quality of hides (1). In addition, certain ticks will cause tick paralysis, which is an acute ascending flaccid motor paralysis caused by the injection of a toxin by the tick while feeding. However, the major effects caused by ticks are due to their ability to transmit protozoan, bacterial and viral diseases to livestock, companion animals and humans (16, 17). Ticks are currently considered to be second only to mosquitoes as vectors of human infectious diseases in the world (9, 23). A number of bacterial zoonotic infectious diseases (2) such as anaplasmosis, ehrlichiosis, and lyme borreliosis are transmitted by ticks.

Table 1 shows the pathogens transmitted by different species of ticks as *Ixodes* species are the vectors of lyme borreliosis, *Amblyomma americanum* is the vector of *Ehrlichia chaffeensi*, tick identification helping in the diagnosis of disease transmitted with, and misidentification of the may lead to difficult and even wrong disease diagnosis.

**Table 1.** Important tick-borne diseases of humans

**Pathogens**	**Disease**	**Vectors**	**Distribution**	**Reference**
***Borrelia burgdorferi*senso lato**	Lyme borreliosis	*Ixodes ricinus*, *I. pacificus*, *I. scapularis*, *I. hexagonus*	Asia, Europe, North America	17,18,19
***Ehrlichia canis***	Human Ehrlichiosis	*Rhipicephalus sanguineus*	South America, Asia, Africa	19
***Ehrlichia ewingii***	Human ewinigii ehrlichiosis	*Amblyomma americanum*	USA, Africa, Asia	20
***Ehrlichia muris***	Murine splenomegaly	*Haemaphysalis*spp, *Ixodes* spp	Eurasia	20
***Ehrlichia chaffeensis***	Human monocytic ehrlichiosis	*Amblyomma americanum*	North America	17,18,19
***Ehrlichia ruminantium***	Heartwater in ruminant	*Amblyomma*spp	Africa, Caribbean	20
***Rickettsia conorii***	Mediterranean spotted fever	*Rhipicephalus sanguineus*, *R. turanicus*	Africa, Asia, Europe	17,18,19
***Coxiella burnetii***	Q fever	Many species	Africa, Asia, Europe, North America, Australia	17,18,19
***Rickettsai rickettsii***	Rocky Mountain spotted fever	*Amblyomma americanum*, *Rhipicephalus sanguineus*, *Dermacentor variablis*	North, South and Central America	17,18,19
***Anaplasma phagocytophilum***	Human granulocytic anaplasmosis	*Haemaphysalis concinna*, *H. punctate*, *Ixodes ricinus, I. pacificus*, *I. scapularis Rhipicephalus bursa*	North America, Europe	17,18,19
***Flavivirus***	Tick borne encephalitis	*Ixodes ricinus, Haemaphysalis concinna*, *H. punctate*	Asia, Europe	18,19
***Babesia divergen, B. microti***	Babesiosis	*Ixodes ricinus*, *I. scapularis*	North America, Europe	17,18,19

Even though there is a lack of whole tick genome annotation, molecular techniques in acarology have been made available in the past few years (9), traditionally tick identification has always been based on morphological characteristics. Moreover, to identify the immature tick stages (larvae, nymph and adults) separate keys are normally used (2, 3). In certain situation, damage to tick body parts essential for their identification (such as capitulum and adjacent structures) may occur during removal of attached ticks to their hosts. In addition, bad preservation of tick samples often occurs, leading to incorrect identifications (4). Identification of some tick species like *Rhipicephalus sanguineus* and *Amblyomma cajennense* is difficult due to the fact that they have been classified as a species complex (11, 12). For instance, *A. cajennense* is a complex of 6 species, while *R. sanguineus* group comprises a total of 17 different species. In these complexes, the different species are geographically separated, due to the large geographical range of distribution, and the expected adaptation of tick populations to different environmental conditions (12). Therefore, morphological identification of these species is not sufficient and the further molecular information is needed for a correct species determination (11, 12).

These difficulties may be reduced when using molecular techniques for tick identification (3). Another benefit from molecular techniques is that with those samples that tick DNA integrity has not been compromised, from the total DNA extraction, a collection of different tick-borne pathogens can be detected by molecular methodologies such as conventional PCR, and real-time PCR (4). Therefore, with one single extraction, both the agent and the vector species can be determined (4). Moreover, the availability of genetic sequence will provide the opportunity to study both the vector population diversity, as well as the pathogen they carry, and potentially even their relationships (13). Currently, there is a lack of whole tick genomes readily annotated (https://www.vectorbase.org/), with *Ixodes scapularis* being the only one currently available (https://www.vectorbase.org/organisms/ixodes-scapularis) (13). This lack of information limits the advancement of the development of new molecular methods for the study of these arthropods. One of the limitations is the large size of tick genomes (15). For instance, the average haploid genome of the tick *I. scapularis* has been calculated at 2262Mbp in length, while the *A. americanum* is around 3108Mbp. If we compare this to the human genome, tick genomes tend to be twice as bigger as the human genome. Part of the difference in size is due to the presence of non-coding regions with extended tandem repeats, that difficulty significantly the sequencing and annotation of those genomes (15). Consequently, different molecular markers have been traditionally used for the phylogeny of ticks (8). Those include the nuclear ribosomal genes 18S rDNA, 28S rDNA and ITS-1, 2 rDNA as well as mitochondrial genes such as 16S, 12S, COI, COIII) rDNA (8, 9).

El-Fiky and El Kammah (3) and Chitimia (4) successfully used the internal transcribed spacer (ITS) for the identification of *Dermacentor marginatus*, *Ixodes ricinus*, *Haemaphysalis*, *Boophilus*, and *Rhipicephalus sanguineus* tick species. On the other hand, the 16S rDNA were able to construct the phylogeny of both hard and soft ticks (6, 8, 9). 16S rDNA were used in molecular classification of *metastriate* ticks (*Dermacentor*, *Amblyomma* and *Rhipicephalus* respectively (10, 13).

Since many tick identification studies are based on the analysis of 16S rDNA, 12S rDNA and ITS-1, 2 rDNA genes, the objective of the present study was to compare the performance of these molecular markers of common use for the identification of ticks, under a diagnostic laboratory environment (13).

## Materials and Methods

### Tick sample collection

Overall, 192 specimens were utilized in this study. This collection was divided into two groups, group A comprises 59 ticks (larvae, nymph, males and engorged females) obtained from Texan citizens through the tick testing service provided by the Lyme Laboratory at Texas A and M University, from January 2014 to August 2015. On the other hand Group, B contains 133 tick pools collected from wildlife through an ecology project conducted in collaboration with Dr. Castro-Arellano at Texas State University (Table 2). For better DNA extractions, larvae and nymphs were analyzed in pools, sometimes the pools contain one larvae and other up to 50 larvae of the same tick species collected from the same location in the same sampling effort. Nymphs, on the other hand, were pooled in groups of from one till 15 specimens, following the same strategy described for larvae for optimal DNA extraction. For the majority of these ticks, the morphological identification was not enough to determine the species, mostly due to either bad storage of samples or loss of mouth-parts while removal the tick from the host. These ticks were immersed in 70% ethanol solution and then processed for DNA extraction and molecular identification using 16S rDNA PCR specific primers and sequencing the PCR product.

**Table 2.** Ticks samples utilized in this study according to their distribution and stages

**Distribution**	**Adult female**	**Adult male**	**Nymph**	**Larvae**
**Arroyo, Colorado**				1
**Mason Mountain**			2	
**Brazos County, Texas**	24	1	1	
**Jefferson County**	2	2	1	
**Texas**	2			
**San Antonio, TX**			3	
**Gus Engeling WMA**			2	5
**Tejas Ranch**				6
**Chaparral WMA**			4	10
**Las Palomas WMA-Arroyo Colorado Unit**			86	52
**Total**	28	3	99	74

### DNA Extraction

DNA was extracted from the tick samples using Wizard® SV Genomic DNA Purification kit (Promega Corporation, Madison, WI) following manufacturer’s recommendations with modifications. Briefly, ticks were incubated for 10min at 70 °C in 200µl of Nuclei Lysis Solution, plus 50µl of 0.5M EDTA, 40 µl of a 20mg/ml Proteinase K solution, and 5µl RNase A Solution. After the initial digestion and for the optimal extraction of DNA from the arthropods, adult individual ticks were homogenized utilizing the bead mill Bead Ruptor 24 (Omni International, Inc., Kennesaw, GA), un-engorged ticks were homogenized with 1.4mm ceramic beads while 2.8mm ceramic beads were used with engorged ticks. After homogenization, tubes were centrifuged at 10,000×g to eliminate tick debris. Supernatants were collected and 250µl of Wizard® SV Lysis Buffer was added to each sample and the mixture. The mixture was run through filter columns at 13,000×g for 3min. DNA bound to filter was washed and eluted following manufacturer recommendations. To extract DNA from the tick immature stages (nymphs and larvae) pools of a maximum of 15 nymphs or 50 larvae were made. Specimens received a code indicating the type of pool generated. All immature specimens were stored at –80 °C with 100µl TE buffer for at least one hour. Specimens were homogenized utilizing pestles while the samples were frozen, followed by DNA extraction procedures using the prepGEM™ (ZyGem Ltd., New Zeland) Insect DNA Extraction kit following manufacturer’s recommendations. Briefly, tick samples were mixed with ultra-pure water, 10x buffer provided in the kit, and 1µl of the prepGEM™ enzyme (ZyGem Ltd., New Zeland). The mixture was incubated at 75 °C for 15min followed by incubation at 95 °C for 15min. The extracted DNA concentration and purity were measured using a NanoDrop, and stored at –20 until use.

### Molecular identification of ticks based on 16S rDNA Gene, 12S rDNA and ITS-1 and 2 rDNA Genes

The tick 16SrDNA was amplified from each specimen studied using conventional PCR methodologies and utilizing primers (6) (Table 3) and AccuPrime™ SuperMix (Quanta BioscienceInc., Gaithersburg, Maryland). The PCR was run following the cycling condition: initial denaturation at 95 °C for 5min followed by 10 cycles of 92 °C for 1min, 48 °C for 1min and 72 °C for 90sec, this step was followed by additional 32 cycles of 92 °C for 1min, 54 °C for 35sec and 72 °C for 90sec, this was followed by a final extension at 72 °C for 7min (5). The amplification products from 16S rDNA were separated on 1.6% agarose gel containing 0.4µg/ml of ethidium bromide (Bio-Rad Laboratoies Inc., Hercules, CA) at 90 volts for 40–60min, and imaged using ChemiDoc touch imaging system (Bio-Rad Laboratoies Inc., Hercules, CA). Positive bands were excised from the gel and purified using the Wizard® SV Gel and PCR clean-up system (Promega Corporation, Madison WI) following manufacturer’s recommendations. The purified products were sent for sequencing (Eton Biosciences, San Diego, CA). Sequences were analyzed through BLAST® in MacVector 14.0.0 software (MacVector Inc., Cary, NC).

**Table 3.** Primers using in PCR

**Gene**	**F-Primers**	**R-Primers**	**Ref**
**Tick 16S rDNA**	5′-TTGGGCAAGAAGACCCTATGAA-3′	5′-CCGGTCTGAACTCAGATCAAGT-3′	(5)
**Tick 12S rDNA**	5′-GAGGAATTTGCTCTGTAATGG-3′	5′-AAGAGTGACGGGCGATATGT-3′	(21)
**ITS-1rDNA**	5′-TCATAAGCTCGCGTTGATT-3’	5′-AGCTGGCTGCGTTCTTCAT-3’	(3)
**ITS-2rDNA**	5′-CGAGCTTGGTGTGAATTGCA-3′	5′-TCCCATACACCACATTTCCCG-3’	(3)

PCR was performed first using16S rDNA Gene primers. The same samples were also tested using specific primers for first and second internal transcribed spacers (ITS-1 and ITS-2 rDNA) (Table 3) following methodologies (4). The PCR reaction was done using the following cycling condition: initial denaturation at 95 °C for 5min followed by forty cycles of 95 °C for 45sec, 55 °C for 1min and 72 °C for 90sec with a final extraction at 72 °C for 1min. The amplification products were separated on 1.6% agarose gel containing 0.4µg/ml of ethidium bromide (Bio-Rad Laboratoies Inc., Hercules, CA) and the gel was run at 90 volts for 40–60min. Gels were visualized using the ChemiDoc touch imaging system (Bio-Rad Laboratoies Inc., Hercules, CA).

In 12S rDNA, PCR was done following the cycling condition: initial denaturation at 95 °C for 5min followed by forty cycles of 95 °C for 30sec, 40 °C for 30sec and 72 °C for 30sec, with a final extraction at 72 °C for 5 min. The amplification products were separated and visualized as mentioned before on 1.6% agarose gel containing 0.4µg/ml of ethidium bromide (Bio-Rad Laboratoies Inc., Hercules, CA) and the gel was run at 90 volts for 40–60min. Gels were visualized using the ChemiDoc touch imaging system (Bio-Rad Laboratoies Inc., Hercules, CA).

### Sequence analysis

Positive bands were excised from the gel and purified using the Wizard® SV Gel and PCR clean-up system (Promega Corporation, Madison WI) following manufacturer’s recommendations. The purified products were sent for sequencing (Eton Biosciences, San Diego, CA). Sequences were analyzed through BLAST® using MacVector 14.0 software (MacVector Inc., Cary, NC).

## Results

Overall, 192 ticks were analyzed from different developmental stages (larvae, nymph, males and engorged females) collected through the state of Texas. In this collection, the tick specimens were identified based on the16S rDNA PCR products as *Dermacentor albipictus*, *D. variabilis*, *Amblyomma americanum*, *Ixodes scapularis*, *A. cajennense Rhipicephalus sanguineus* and *Carios capensis* (Table 4), the GenBank accession numbers from KX673167 to KX673180.

**Table 4.** Ticks samples utilized in this study according to their distribution and species

**No.**	**Samples name**	**Tick species**	**Location**	**Developmental stage**
**1**	ARCO1	*Dermacentor variabilis*	Arroyo, Colorado	Larvae
**2**	MMSL7	*Amblyomma americanum*	Mason Mountain	Nymph
**3**	MMSL8	*Amblyomma americanum*	Mason Mountain	Nymph
**4**	CETX-2	*Ixodes scapularis*	Texas	adult female
**5**	LPTX-1	*Rhipicephalus sanguineus*	Brazos County, Texas	adult female
**6**	BAS115	*Amblyomma cajennense*	Brazos County, Texas	adult female
**7**	BBLC1	*Amblyomma americanum*	Brazos County, Texas	adult female
**8**	RITX-1	*Ixodes scapularis*	Jefferson County	adult female
**9**	WOTX-1	*Ixodes scapularis*	Brazos County, Texas	adult female
**10**	SG-1	*Ixodes scapularis*	Brazos County, Texas	adult female
**11**	NSTX-1	*Ixodes scapularis*	Texas	adult female
**12**	BETX-20	*Ixodes scapularis*	Jefferson County	adult female
**13**	SAT-88	*Rhipicephalus sanguineus*	San Antonio, TX	Nymph
**14**	KTTX-5	*Dermacentor variabilis*	Brazos County, Texas	adult male
**15**	KTTX-6	*Dermacentor variabilis*	Brazos County, Texas	adult female
**16**	BAS-183	*Amblyomma maculatum*	Brazos County, Texas	adult female
**17**	BAS-125	*Dermacentor variabilis*	Brazos County, Texas	adult female
**18**	BAS-126	*Dermacentor variabilis*	Brazos County, Texas	adult female
**19**	BAS-127	*Dermacentor variabilis*	Brazos County, Texas	adult female
**20**	BAS-128	*Amblyomma maculatum*	Brazos County, Texas	adult female
**21**	BAS-129	*Dermacentor variabilis*	Brazos County, Texas	adult female
**22**	BAS-216	*Amblyomma maculatum*	Brazos County, Texas	adult female
**23**	BAS-124	*Dermacentor andersoni*	Brazos County, Texas	adult female
**24**	SAT-97	*Rhipicephalus sanguineus*	San Antonio, Texas	Nymph
**25**	SAT-101	*Rhipicephalus sanguineus*	San Antonio, Texas	Nymph
**26**	MTX1	*Ixodes scapularis*	Brazos County, Texas	adult female
**27**	MTX3	*Ixodes scapularis*	Brazos County, Texas	adult female
**28**	MTX4	*Ixodes scapularis*	Brazos County, Texas	adult female
**29**	BETX-16	*Ixodes scapularis*	Brazos County, Texas	adult female
**30**	BETX-17	*Ixodes scapularis*	Brazos County, Texas	adult female
**31**	BETX-18	*Ixodes scapularis*	Brazos County, Texas	Nymph
**32**	BETX-19	*Ixodes scapularis*	Brazos County, Texas	adult female
**33**	THREAD1	*Ixodes scapularis*	Brazos County, Texas	adult female
**34**	CSTX1	*Ixodes scapularis*	Brazos County, Texas	adult female
**35**	CVM11	*Dermacentor albipictus*	Brazos County, Texas	adult female
**36**	CETX-2	*Dermacentor albipictus*	Brazos County, Texas	adult female
**37**	TJM 305-5	*Carios capensis*	Chaparral WMA	Larvae
**38**	TJM 448-514	*Carios capensis*	Las Palomas WMA - Arroyo Colorado Unit	50 Larvae
**39**	TJM 182-1	*Carios capensis*	Chaparral WMA	Nymph
**40**	TJM 448	*Carios capensis*	Las Palomas WMA - Arroyo Colorado Unit	48 Nymphs
**41**	TJM 216.1	*Carios capensis*	Chaparral WMA	Nymph
**42**	TJM 308-12	*Carios capensis*	Chaparral WMA	3 Larvae
**43**	TJM 596	*Dermacentor variabilis*	Las Palomas WMA - Arroyo Colorado Unit	38 Nymphs
**45**	TJM112	*Dermacentor variabilis*	Gus Engeling WMA	2 Larvae
**46**	TJM 355	*Dermacentor variabilis*	Chaparral WMA	Nymph
**47**	TJM 440	*Dermacentor variabilis*	Las Palomas WMA - Arroyo Colorado Unit	1 Larvae
**48**	TJM 308-18	*Dermacentor variabilis*	Chaparral WMA	3 Larvae
**49**	TJM 140-3	*Amblyomma inornatum*	Gus Engeling WMA	3 Larvae
**50**	TJM 139	*Amblyomma inornatum*	Gus Engeling WMA	2 Larvae
**51**	TJM 529	*Dermacentor variabilis*	Las Palomas WMA - Arroyo Colorado Unit	1 Larvae
**52**	TJM 216	*Amblyomma maculatum*	Chaparral WMA	Nymph

### Comparison between 16S rDNA Gene, 12S rDNA Gene and (ITS-1, 2) rDNA Genes

**Fig. 1.**
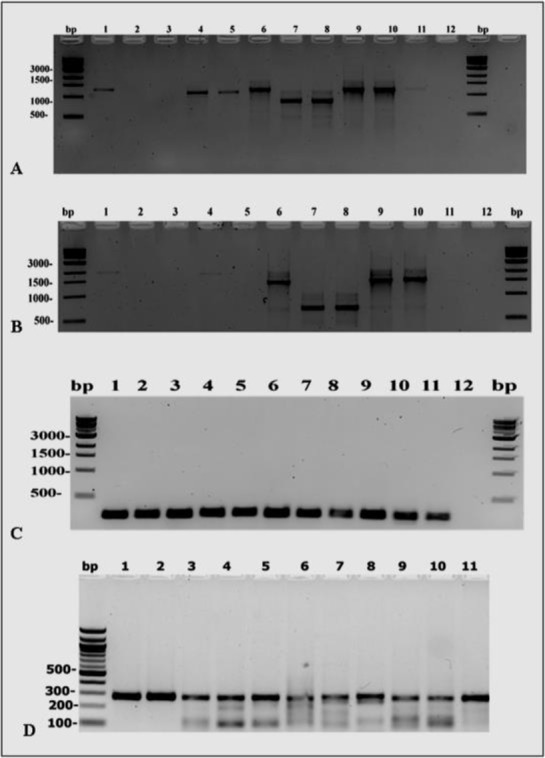
PCR amplification utilizing Tick samples of different species using: (A) the ITS-2PCR reaction (B): ITS-1 PCR reaction and (C): 16S r DNA Gene, (D)12SrDNA DNA ladder is located on the left and right sides of the gel, fragment sizes are represented in base pairs (bp), 1: *Rhipicephalus sanguineus* sample (SAT 97), 2: *Rhipicephalus sanguineus* sample (SAT88), 3: *Rhipicephalus sanguineus* sample (SAT 101), 4: *Amblyomma americanum* sample (MMSL5), 5: *Amblyomma americanum* sample (MMSL9), 6 *Amblyomma cajennense (*BAS 115 TICK): 7: *Ixodes scapularis* sample (CSTX-2), 8: *Ixodes scapularis* sample (CSTX-3), 9: *Dermacentor albipictus* sample(CVM11), 10: *Dermacentor albipictus* sample(CETX-2), 11: *Dermacentor* variabilis sample(ARCO1) and 12: negative control.

In order to evaluate which genetic marker perform best under diagnostic conditions, comparison between the amplification of four genes 16S rDNA, 12S rDNA, ITS1 and ITS2 rDNA genes was done. Positive bands from 16S rDNA, 12S rDNA PCR were excised from the gel and cleaned and submitted for sequencing. Sequences were analyzed through BLAST® in MacVector 14.0.0 software (MacVector Inc., Cary, NC).

in spite of the samples number are not representative, but changing in the tick PCR amplification of the four genes was observed as good amplification for the samples were observed as both 16S rDNA, 12S rDNA Gene showing good PCR amplification in all the sample but (ITS-1, 2) rDNA Genes failed to amplify some samples species, (Fig. 1).

The samples utilized were *Dermacentor albipictus* (CVM11, CETX-2), *Dermacentor variabilis* (ARCO1), *Amblyomma americanum* (MMSL5, MMSL9), *Ixodes scapularis* (CSTX-2, CSTX-3), *Amblyomma cajennense* (BAS 115) and *Rhipicephalus sanguineus* (SAT 88, SAT 97, and SAT 101).

In ITS-1PCR, one of the three samples of *Rhipicephalus sanguineus* (SAT 101), two samples of *Amblyomma americanum* (MMSL5, MMSL9), *Ixodes scapularis* samples CSTX-2, CSTX-3) and *Dermacentor albipictus* samples (CVM11, CETX-2) were amplified, but there were no amplification in *Dermacentor variablis* (ARCO1), *Amblyomma cajennense* (BAS 115) and the other two samples of *R. sanguineus* (SAT 88, SAT 97).

ITS-2 PCR, one of the three samples of *Rhipicephalus sanguineus* (SAT 101), two samples of *Amblyomma americanum* (MMSL5, MMSL9), *Dermacentor variablis* (ARCO1), *Amblyomma cajennense* (BAS 115), *Ixodes scapularis* samples CSTX-2, CSTX-3) and *Dermacentor albipictus* samples (CVM11, CETX-2) were amplified, but the two samples of *R. sanguineus* (SAT 88, SAT 97) failed to amplify.

### Phylogenetic analysis

**Fig. 2.**
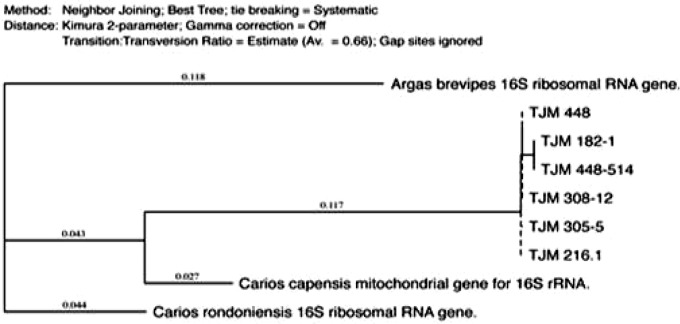
The phylogenetic analysis was constructed using neighbor joining method, to construct the tick phylogenetic tree of some of soft tick species sequences from the Genbank and our sequences samples are included based on 16S r DNA sequences

**Fig. 3.**
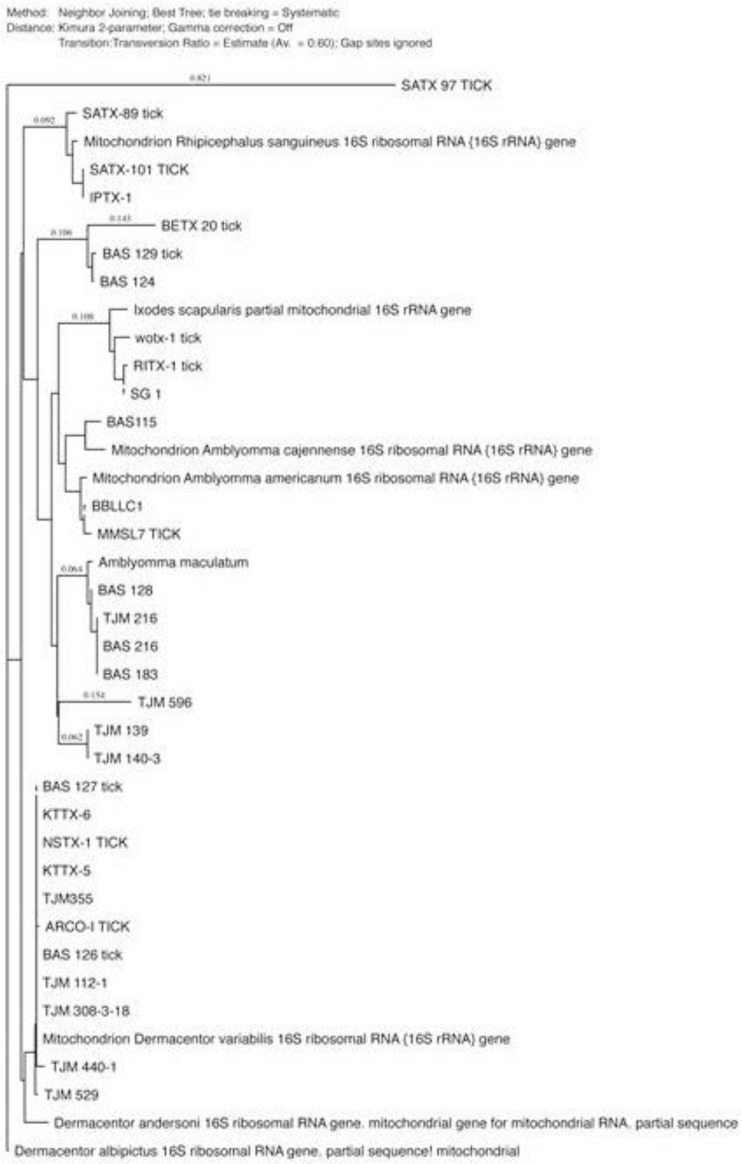
The phylogenetic analysis was constructed using neighbor-joining method, to construct the tick phylogenetic tree of some of hard tick species sequences from the Genbank and our sequences samples are included based on 16S r DNA sequences

**Fig. 4.**
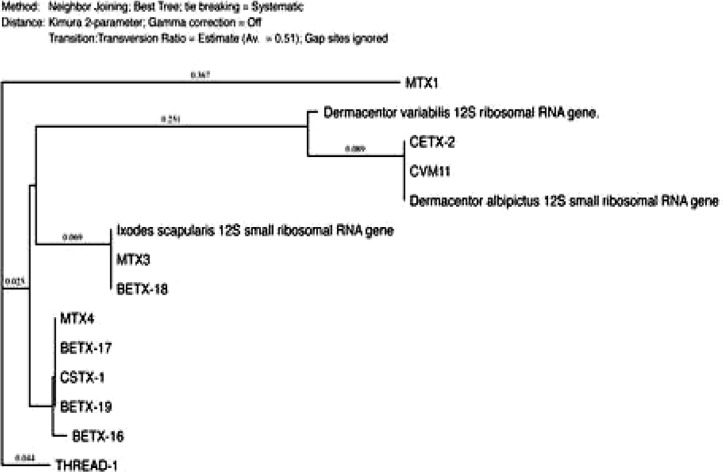
The phylogenetic analysis was constructed using neighbor-joining method, to construct the tick phylogenetic tree of some of hard tick species sequences from the Genbank and our sequences samples are included based on 12S r DNA sequences

The phylogenetic analysis was performed using MacVector 14.0 software (MacVector Inc., Cary, NC) and the tree was constructed using neighbor-joining (NJ) methods (Figs. 2–4). The low degree of sequences variation observed within most of the species of the soft and hard tick trees based on the 16S rDNA since they all share the same ancestor. Nevertheless, there is one sequence in the hard tick population studied *Rhipicephalus sanguineus* that show higher variation.

## Discussion

The present studies aimed to present good molecular marker for tick identification based on DNA sequences to solve the problems with morphological tick identification, and sometimes the morphological identification is not enough for detect the species so amplification of the16S rDNA using it as a methods for tick genetic identification, and comparison between the amplification of the the16S rDNA, 12S rDNA, ITS-1 rDNA and ITS-2 rDNA for the same samples species.

16S rDNA and 12S rDNA Genes are a mitochondrial ribosomal DNA gene, mtDNA considered one of the most commonly used genes for molecular identification of ticks due to the fact that it is relatively easy to work with them due to their higher copy number (8). In addition, mtDNA sequences are a good phylogenetic marker mostly for groups of organisms, diverged relatively, since mtDNA has a higher rate of base substitution than most nuclear markers (9). The problem for the mitochondrial gene is it can transfer to the nucleus leading to error in the phylogeny after the amplification and sequencing (8). The difference between the 16S rDNA and 12S rDNA Genes that the evolution is faster in 12S rDNA Genes (8).

Regarding tick species, 16S rDNA was used and succeeded to construct phylogeny of both hard and soft ticks (6, 7, 8) and 16S rDNA is useful in constructing their tick phylogenetic tree, but there is a problem associated with 16S rDNA is that using this gene alone is not sufficient for getting full resolution for the tree so the best way to solve it accompanied it with another gene like 12S rDNA (6, 8). We utilized these genes for diagnostic purpose only not for phylogeny, and the problems are usually associated with the phylogeny.

Overall, 192 tick samples (larvae, nymph, males and engorged females) were evaluated using16S rDNA PCR and 12S rDNA the PCR positive bands have to be sequencing. The sequencing analysis determined that the tick species collected in the study were: *Amblyomma americanum*, *Dermacentor albipictus*, *Ixodes scapularis*, *D*. *variabilis*, *A*. *cajennense*, *Carios capensis* and *Rhipicephalus sanguineus* (Table 3). Therefore, using 16S rDNA and 12S rDNA are good in molecular tick identification of all these species utilized in our study.

The first and the second internal transcribed spacers region of the nuclear ribosomal gene cluster (ITS-1, ITS-2), consist of three genes 18SrDNA, 5.8SrDNA and 28SrDNA. These three rDNA genes are transcribed making a single transcript of RNA separated by the ITS-1 and ITS-2 regions (7). (ITS-1, ITS-2) considered the fastest evolving DNA genes (9). Because of these facts, the ITS-1, 2 rDNA are not good in amplification of some of the tick species in our study, they failed to amplify some tick species as mentioned before. Internal transcribed spacer was used successfully for the identification of *Dermacentor marginatus*, *Ixodes ricinus*, *Haemaphysalis*, *Boophilus*, and *Rhipicephalus sanguineus* tick species (3, 4).

Nevertheless, the problem with internal transcribed spacer is that genes are evolving rapidly so in some species they failed to amplify as reported before in ticks (8). ITS-2 was utilized for identification Iranian hard tick and ITS-2 failed to amplify some of his samples. Therefore, these markers were mostly useful to study close related species (10). Even some of our samples are closely related to each other as *Rhipicephalus* species and *Dermacentor* and it failed to amplify some of them, therefore, it is better to clone the PCR products and work with it as haplotypes, not individuals make the studies more expensive (8).

## Conclusion

Molecular tick identification will help and improve the disease diagnosis and choosing good genetic marker for diagnosis purpose as 16S rDNA and 12S rDNA markers is good as they give good amplification for our sample species, in spite of using (ITS-1, ITS**-**2) are good for tick molecular identification for very closely related species but with our few samples even they are not representative samples it did not work with the closely related tick species.
